# Human Antibody V_H_ Domains Targeting GPNMB and VCAM-1 as Candidate Therapeutics for Cancers

**DOI:** 10.1021/acs.molpharmaceut.3c00173

**Published:** 2023-04-17

**Authors:** Xiaojie Chu, Wei Li, Margaret G. Hines, Ilya Lyakhov, John W. Mellors, Dimiter S. Dimitrov

**Affiliations:** † Center for Antibody Therapeutics, Division of Infectious Diseases, Department of Medicine, University of Pittsburgh Medical School, Pittsburgh, Pennsylvania 15261, United States; ‡ Comp IL, LLC, Carnegie, Pennsylvania 15106, United States; § Abound Bio, Pittsburgh, Pennsylvania 15219, United States

**Keywords:** therapeutic antibody, V_H_ domain, human GPNMB (DC-HIL, Osteoactivin), human VCAM-1, DbTE (Domain based bispecific T cell engager)

## Abstract

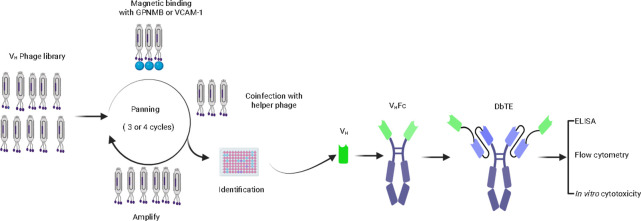

The elevated expression of GPNMB and VCAM-1 has been observed in many cancers including breast cancer, melanoma, and prostate cancers. Such overexpression of GPNMB and VCAM-1 has been associated with poor prognosis and increased cancer metastasis. Thus, GPNMB and VCAM-1 are potential targets for immunotherapies across multiple cancers. In this study, two high-affinity specific human V_H_ domain antibody candidates, 87 (GPNMB) and 1B2 (VCAM-1), were isolated from our in-house proprietary phage-displayed human V_H_ antibody domain libraries. The avidity was increased after conversion to VH-Fc. Domain-based bispecific T-cell engagers (DbTE) based on these two antibodies combined with the anti-CD3ε OKT3 antibody exhibited potent killing against GPNMB and VCAM-1-positive cancer cells, respectively. Hence, these two domain antibodies are promising therapeutic candidates for cancers expressing GPNMB or VCAM-1.

## 1 Introduction

Glycoprotein nonmetastatic melanoma protein B (GPNMB), also known as osteoactivin (OA), dendritic cell-heparin integrin ligand (DC-HIL), or hematopoietic growth factor inducible neurokinin-1 type (HGFIN), is a type 1 transmembrane protein. It is highly expressed in many tumors including melanoma,^1^ prostate cancer,^2^ lung cancer,^3,4^ bladder cancer,^5^ breast cancer,^6,7^ and gliomas.^8^ This increased expression is often associated with poor prognosis and overall survival in affected patients. In the tumor microenvironment, GPNMB functionally promotes tumor progression and invasion, cell adhesion and differentiation,^9^ endothelial cell recruitment, and metastasis through multiple mechanisms including interaction with syndecan-4 to block the proliferation and activation of T cells,^10^ interaction with integrin by the extracellular domain of GPNMB to induce trans-endothelial migration,^11^ interaction with the C-terminus of EGFR (epidermal growth factor receptor) to activate EGFR pathway, which further activate STAT3 (signal transducer and activator of transcription 3) signaling and promote cancer metastasis,^12,13^ and activation of MMP-3 (matrix metallopeptidase 3), which is involved with cancer cell migration, invasion, and inflammation.^14^

Vascular cell adhesion molecular 1 (VCAM-1), also named CD106, shows similar behavior in cancerous tissues. VCAM-1 is expressed on the luminal and lateral side of endothelial cells under inflammatory stimulation.^15^ As an immunoglobulin superfamily member, it plays an important role in the immune surveillance of many diseases. Recently, studies have shown that the elevated expression of VCAM-1 was involved in tumor cell adhesion on endothelium cells related to metastasis.^16,17^ Like GPNMB, this overexpression was associated with poor prognosis in many cancers including breast cancer,^18^ melanoma,^19^ colorectal cancer,^20^ ovarian cancer,^21^ and prostate cancer.^22^ Overall, the high expression of GPNMB and VCAM-1 in cancers and their functional association with cancer cell growth and metastasis make them important targets for development of antitumor therapeutics.

Antibody-based immunotherapies have become increasingly attractive options in cancer treatment due to their high affinity for target proteins and relatively low toxicity compared to other therapies. More than 100 mAbs have been approved by the FDA (Food and Drug Administration) for immunotherapies of many different diseases like autoimmune and inflammatory diseases and cancers.^23^ In recent decades, the field has seen a growing interest in antibody domains used as diagnostics and therapeutics due to their small size, low immunogenicity, and efficient infiltration into solid cancer tissues. These antibody domains/fragments enable treatments to target new epitopes that are not accessible to full-size (IgG) antibodies or large antibody constructs. Recent studies show that variable domain antibodies can even pass through the blood–brain barrier.^24^ Thus, the use of variable domain antibodies may be a powerful tool in the development of cancer immunotherapies.

In our current study, we identified two potent human V_H_ domain antibodies that target GPNMB and VCAM-1. These binders were characterized for their affinity and specificity. The domain-based bispecific T cell engagers (DbTE) constructed by these two binders showed potent killing effects on GPNMB and VCAM-1-expressing cancer cells, respectively. This is the first report of GPNMB or VCAM-1-specific human V_H_ domain antibodies as candidates for cancer immunotherapy.

## 2 Materials and Methods

### 2.1 Panning of High-Affinity V_H_ Domains against GPNMB and VCAM-1 from Large V_H_ Phage Library

Human GPNMB-Fc, VCAM-1-Fc, and VCAM-1-His recombinant proteins were purchased from R&D systems. Human GPNMB-His was purchased from Acro Biosystems. To perform panning antibody candidates against GPNMB and VCAM-1, a large phage-displayed human V_H_ library was used against human IgG1 Fc fused recombinant GPNMB and VCAM-1 separately. The panning was performed as previously described.^25^ The V_H_ phage library was first incubated with 50 μL Protein G magnetic beads (Thermo Fisher Scientific) to remove nonspecific binders. The phage was then blocked with 5% milk and incubated with 5 μg of GPNMB-Fc or VCAM-1-Fc protein. The libraries were then incubated with Protein G magnetic beads to separate the antigen-bound phages. The beads were washed with PBST followed by PBS before directly infection of log phase TG1 *E. coli* cells for phages expression and amplification. Three more rounds of subsequent panning were performed, which reduced the GPNMB-Fc or VCAM-1-Fc concentration one fold each round. After four rounds of panning, 192 individual clones were screened for binding GPNMB-His or VCAM-1-His protein by ELISA.

### 2.2 Expression and Purification of V_H_, V_H_-Fc, and DbTE

To convert V_H_ antibody candidates to V_H_-Fc format, the V_H_ domain was amplified and cloned into the pcDNA-IgG1 Fc vector. For the construction of DbTE, humanized OKT3^26^ was inserted at the C terminal of V_H_ followed by the IgG1 Fc with LALAPG mutation. The expression and purification were performed as previously described.^25^ Briefly, the V_H_-Fc and DbTE were transiently transfected and expressed by the Expi293 expression system and purified by protein A resin (Thermo Fisher Scientific). The V_H_ binder was expressed in the *E. coli* TopF′ expression system and purified on Ni-NTA columns (GE Healthcare Life Sciences).

### 2.3 Biolayer interferometry BLItz

The affinity and avidity of the anti-hGPNMB and anti-hVCAM-1 antibodies were detected by biolayer interferometry BLItz (ForteBio) as described previously.^25^ Briefly, DPBS was used to establish a baseline for 30 s, and streptavidin biosensors were coated with 16.7 μg/mL recombinant GPNMB-Biotin or VCAM-1-Biotin for 2 min. Different doses of V_H_ and V_H_-Fc were used for association and monitored for 2 min to measure the affinity and avidity. Antibody dissociation was monitored in DPBS for 4 min. Antigen-coated biosensors in PBS served as a reference control.

### 2.4 Cell Lines

Expi293 cells (Thermo Fisher Scientific) were maintained in an Expi293 expression medium supplemented with 0.4% penicillin–streptomycin (P/S). 293T cells and SK-MEL-28 cells were purchased from ATCC and maintained in DMEM or EMEM supplemented with 10% FBS and 1% P/S. HuT-78 cells purchased from ATCC were maintained in IMDM supplemented with 20% FBS and 1% P/S. 293T-GPNMB/VCAM-1 cell lines were obtained by transfection with the plasmid containing GPNMB gene or VCAM-1 gene linked with the Zeocin resistant. The stable cell line of 293T-GPNMB/VCAM-1 was selected by using a high concentration of Zeocin antibiotic (500 μg/mL) for 7 days, and stable cells were maintained with 100 μg/mL of Zeocin.

### 2.5 T Cell Isolation

T-cell isolation was performed as previously described.^27^ T cells were isolated from healthy donor’s PBMCs (Zen-Bio) by using the human Pan T cell isolation kit (Miltenyi Biotec) and activated by CD3/CD28 T cell activator Dyna beads (Gibco, Thermo Fisher Scienctific) at 1:1 cell/bead ratio for 48 h. The activated T cells were used for the cytotoxicity assay of the DbTE antibody.

### 2.6 Flow Cytometry

The cell surface expression level of GPNMB and VCAM-1 protein was detected by a commercial antibody. For this, 2 × 10^5^ cells/test were stained with anti-hGPNMB-PE or anti-hVCAM-1-PE antibody (Invitrogen, Thermo Fisher Scienctific) for 30 min at 4 °C. To verify the cell surface binding of the isolated antibody, cells were incubated with 50 nM V_H_-Fc 87, V_H_-Fc 1B2, 1 μM V_H_ 87, or V_H_ 1B2 for 30 min at 4 °C, respectively. Cells were then stained with a secondary antibody, goat antihuman IgG-PE (Sigma-Aldrich) 1:250 for V_H_-Fc, or 2 μL of anti-Flag-APC (Miltenyi Biotec) for V_H_. Irrelevant V_H_-Fc and V_H_ were used as isotype controls.

### 2.7 Western Blot

Total proteins were extracted from 293T, Sk-mel-28, and HuT-78 cells by RIPA buffer containing 1× protease inhibitors (Thermo Fisher Scienctific). 20 μg of total proteins of each sample was loaded into a 4 to 12% Bis-Tris mini protein gel (Thermo Fisher Scienctific). Proteins were then transferred to a 0.2 μm PVDF membrane (BioRad). The membrane was blocked in 5% no-fat milk for 1 h at room temperature, followed by incubation with rabbit antihuman GPNMB antibody at 1:1000 dilution (Cell Signaling Technology), mouse antihuman VCAM-1 antibody at 2 μg/mL (R&D systems), or β-Actin antibody at 1:1000 (Cell signaling technology) at 4 °C overnight. After three washes with TBST, the membranes were incubated with goat antirabbit HRP secondary antibody at 1:3000 dilution or goat antimouse HRP secondary antibody at 1:5000 dilution (Thermo Fisher Scienctific) for 1 h at room temperature. The membranes were visualized by using ECL Western blot substrate (Thermo Fisher Scienctific) and imaged by a ChemiDoc XRS+ imaging system (Bio-Rad).

### 2.8 Cytotoxicity Assays

The cell cytotoxicity of anti-GPNMB and anti-VCAM-1 DbTE was measured by LDH-Glo cytotoxicity assay kit (Promega) following the manufacturer’s instructions. Target cells (1 × 10^4^ cells/well) and activated T cells were seeded in a 96-well plate at an E/T ratio 10:1, mixed with serially diluted DbTE antibodies in a growth medium, and incubated for 24 h at 37 °C in 5% CO_2_ humidified atmosphere. The final volume was 100 μL/well. The cell supernatant was diluted 20-fold and incubated for 50 min for LDH assay setup. The calculation of relative % cytotoxicity is as follows: relative % cytotoxicity = 100 × (Experimental LDH release – Target and effector cell only)/(target and effector maximum LDH release control – Target and effector cell only).

### 2.9 Statistical Analysis

Statistical analyses were performed by GraphPad Prism. Differences were considered statistically significant when *p* < 0.05. Significance was tested using two-way ANOVA, followed by Tukey’s multiple comparisons tests. ****, *p* < 0.0001, **, *p* < 0.01, *, *p* < 0.05.

## 3 Results

### 3.1 Characterization of Selected High-Affinity V_H_ Antibodies against Human GPNMB or VCAM-1

**Figure 1:**
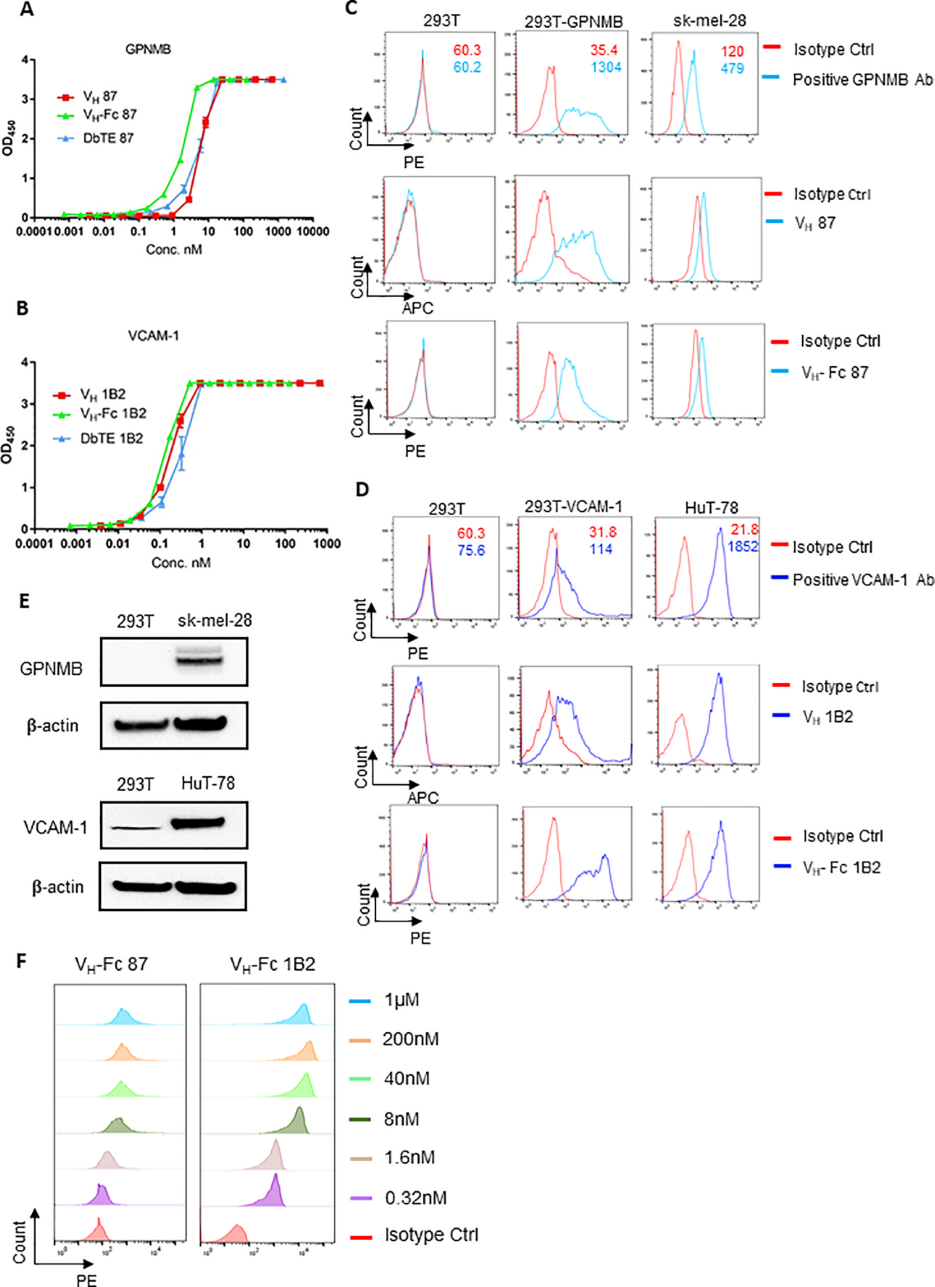
Specificity of V_H_/V_H_-Fc with targets on cell surface. (A) GPNMB binders binding to recombinant human GPNMB measured by ELISA. (B) VCAM-1 binders binding to recombinant human VCAM-1 measured by ELISA. Experiments were performed in duplicate. (C) Detection of cell surface expression of GPNMB on 293T, 293T-GPNMB, and sk-mel-28 by commercial antihuman GPNMB or the V_H_/V_H_-Fc 87 binders. (D) Detection of cell surface expression of VCAM-1 on 293T, 293T-VCAM-1, and HuT-78 cells by commercial antihuman VCAM-1 antibodies or the V_H_/V_H_-Fc 1B2 binders. (E) Verification of GPNMB and VCAM-1 expression on 293T, sk-mel-28, and HuT-78 cells. (F) Dose-dependent cell surface binding of V_H_-Fc 87 on 293T-GPNMB cells (Left) and V_H_-Fc 1B2 on 293T-VCAM-1 cells (Right).

To pan specific antibodies against human GPNMB and VCAM-1, a large phage-displayed human V_H_ library was used. After four rounds of panning, 10 binders for GPNMB and 15 binders for VCAM-1 from 192 clones were identified. Among these, one V_H_ antibody of each target was identified and selected based for its affinity, specificity, and desirable functional properties. The anti-GPNMB antibody was designated as V_H_ 87 and the anti-VCAM-1 antibody was designated as V_H_ 1B2. The EC_50_ of V_H_ 87 and V_H_ 1B2 were 6.1 ± 0.09 nM and 0.2 ± 0.01 nM, respectively (Figure 1A,B). The equilibrium dissociation constant (*K*_D_) values for binding to rhGPNMB or rhVCAM-1 were 13.4 nM and 2.8 nM, respectively, as determined by BLItz (Table 1). To extend the half-life of these antibodies, the two V_H_ binders were converted to a V_H_-Fc format by inserting IgG1 Fc in the C terminal of V_H_. The EC_50_ values of V_H_-Fc 87 and V_H_-Fc 1B2 were 1.8 ± 0.08 nM and 0.13 ± 0.003 nM, respectively (Figure 1A,B). The equilibrium dissociation constant (*K*_D_) values of V_H_-Fc 87 and V_H_-Fc 1B2 as measured by BLItz were 9.3 nM and 1.9 nM, respectively (Table 1).

**Table 1:** BLItz Results of Human GPNMB and VCAM-1 Antibodies

Antibody	*k*_on_ (M^–1^ s^–1^)^1^	*k*_off_ (s^–1^)^1^	*K*_D_ (nM)^1^
V_H_ 87	1.2 × 10^5^ ± 9.8 × 10^2^	1.7 × 10^–3^ ± 4.5 × 10^–5^	13.4
V_H_-Fc 87	1.3 × 10^5^ ± 1.9× 10^3^	1.2 × 10^–3^ ± 3.7 × 10^–5^	9.3
DbTE 87	1.2 × 10^5^ ± 1.2 × 10^3^	3.0 × 10^–4^ ± 1.8 × 10^–5^	2.6
V_H_ 1B2	1.3 × 10^5^ ± 1.2 × 10^3^	3.7 × 10^–4^ ± 1.4 × 10^–5^	2.8
V_H_-Fc 1B2	2.3 × 10^5^ ± 3.8 × 10^3^	4.6 × 10^–4^ ± 2.2 × 10^–5^	1.9
DbTE 1B2	2.9 × 10^5^ ± 3.1 × 10^3^	3.2 × 10^–5^ ± 1.0 × 10^–5^	0.1

1 Mean kinetic rate constants (*k*_on_, *k*_off_) and equilibrium dissociation constants (*K*_D_ = *k*_off_/*k*_on_) were determined from curve fitting analyses of BLItz results.

To verify the specificity of the two binders to antigens expressed on the cell surface, we first tested the surface expression of GPNMB or VCAM-1 on parental 293T cells, 293T cells isogenically expressing GPNMB (293T-GPNMB) or VCAM-1 (293T-VCAM-1), and cancer cell lines sk-mel-28 (human melanoma cell line) and HuT-78 (human cutaneous T-lymphocyte cancer cell line), which intrinsically express GPNMB or VCAM-1, respectively. The cells were tagged with a commercial antihuman GPNMB antibody and antihuman VCAM-1 antibody. Among these cell lines, 293T-GPNMB cells (MFI of isotype versus positive GPNMB Ab is 35.4 vs 1304) and sk-mel-28 cells (MFI of isotype vs positive GPNMB Ab is 120 vs 479) moderately expressed GPNMB. 293-VCAM-1 cells (MFI of isotype vs positive VCAM-1 Ab is 31.8 vs 114) and HuT-78 cells (MFI of isotype vs positive Ab is 21.8 vs 1852) showed a high expression level of VCAM-1. There was no obvious expression of GPNMB and VCAM-1 on 293T cells (MFI of isotype vs GPNMB/VCAM-1 positive Ab is 60.3 vs 60.2/75.6) as observed by flow cytometry testing (Figure 1C,D). The intrinsic GPNMB and VCAM-1 expressions on 293T cells, sk-mel-28 cells, and HuT-78 cells were further verified by Western Blot using commercial antibodies (Figure 1E). It should be noted that 293T cell shows low expression of VCAM-1 when tested by WB, but this expression was not detectable by flow cytometry. Next, we tested the binding specificity of our newly identified binders on the above cell lines. Results showed that both V_H_ 87 and V_H_-Fc 87 specifically bound to 293T-GPNMB and sk-mel-28 cells but not to GPNMB-negative 293T cells (Figure 1C). Similarly, V_H_ 1B2 and V_H_-Fc 1B2 specifically bound to 293T-VCAM-1 and HuT-78 cells, but not to 293T cells, despite the low VCAM-1 expression level described above (Figure 1D). Moreover, the V_H_-Fc 87 and 1B2 binders bound to the 293T-GPNMB and 293T-VCAM-1 in a concentration-dependent manner, respectively. And the EC_50_ value was 6.6 ± 0.99 nM for V_H_-Fc 87 and 7.3 ± 3.34 nM for V_H_-Fc 1B2 (Figure 1F).

Taken together, the specific binding to membrane-bound GPNMB and VCAM-1 by each binder warrants further development as DbTEs for immunotherapy against GNNMB and VCAM-1 overexpressing cancers.

### 3.2 Candidate DbTEs Shows Potent Cytotoxicity against GPNMB or VCAM-1 Expressing Cells

**Figure 2:**
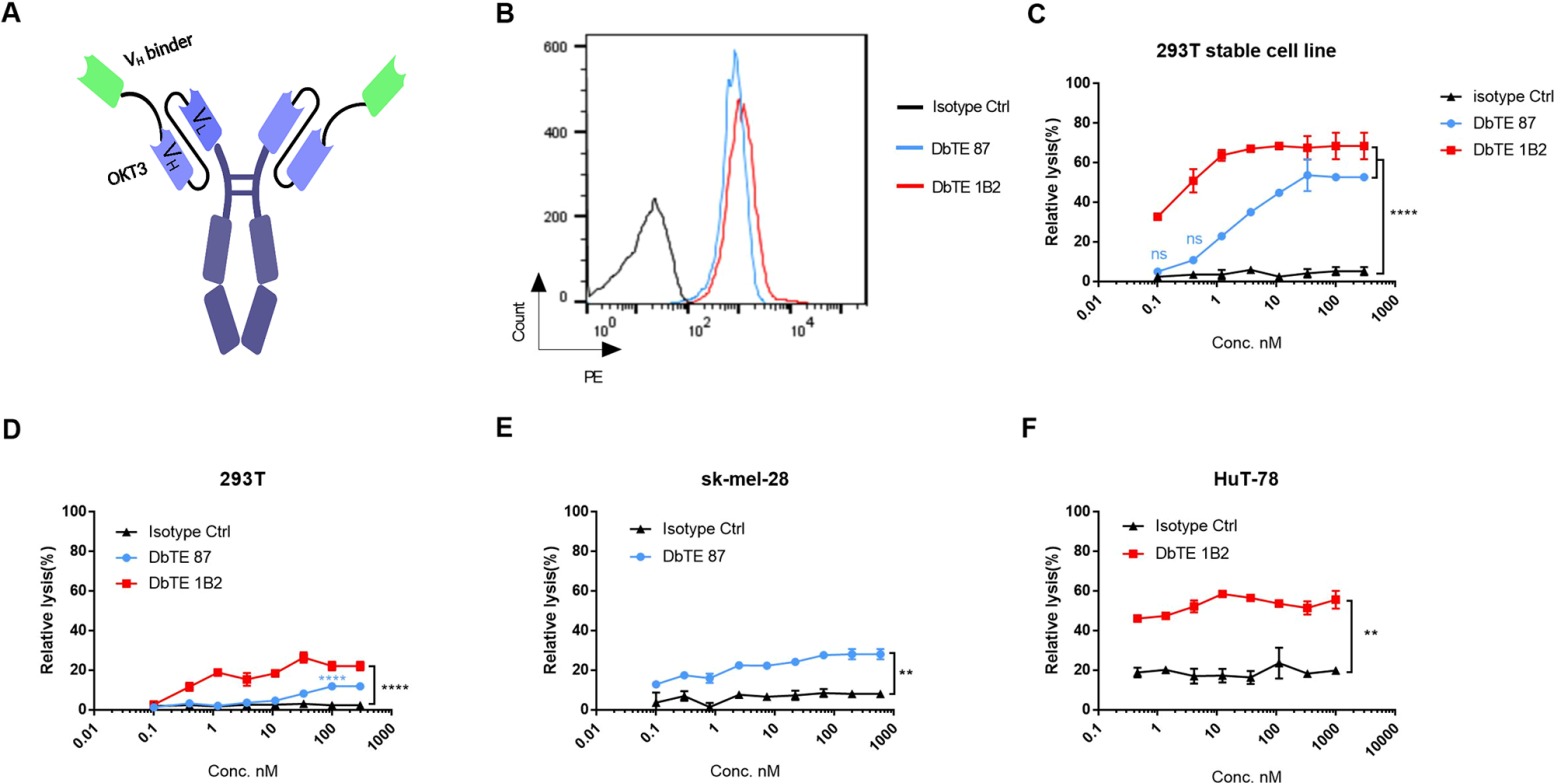
*In vitro* cytotoxicity of T cells to GPNMB-expressing cells or VCAM-1-expressing cells by anti-GPNMB DbTE or anti-VCAM-1 DbTE. (A) Schematic design of domain-based bispecific T cell engager (DbTE). (B) DbTE binding effects with T cells detected by flow cytometry. (C, D), Percent relative lysis of (C) 293T-GPNMB and 293T-VCAM-1, (D) 293T cells by T cells mediated by DbTE 87 and DbTE 1B2, respectively. (E) Percent lysis of GPNMB positive sk-mel-28 cancer cells by T cells mediated by DbTE 87. (F) Percent lysis of VCAM-1 positive HuT-78 cancer cells by T cells mediated by DbTE 1B2; T cells and target cells were added at an E/T ratio of 10:1 and simultaneously treated with serially diluted DbTE antibodies for 24 h. The luminescence signal was detected and used to calculate the percent relative lysis. Experiments were performed in duplicate. Values were reported as the mean of percent relative lysis ± SD. Significance was tested by using two-way ANOVA, followed by Tukey’s multiple comparisons test. ****, *p* < 0.0001, **, *p* < 0.01.

To assess the cell cytotoxicity of GPNMB and VCAM-1 binder-based bispecific T cell engagers, we designed a domain-based bispecific antibody (DbTEs). To construct these DbTEs and extend its half-life, humanized anti-CD3 antibody OKT3 single chain variable fragment (scFv) fused with IgG1 Fc was inserted in the C-terminal of V_H_ 87 or V_H_ 1B2 (Figure 2A). The EC_50_ of DbTE 87 and DbTE 1B2 were 5.4 ± 0.3 nM and 0.3 ± 0.02 nM, respectively (Figure 1A,B). The equilibrium dissociation constant (*K*_D_) values of DbTE 87 and DbTE 1B2 as measured by BLItz were 2.6 nM and 0.1 nM, respectively (Table 1). The specific binding of DbTE 87 and 1B2 with T cells was verified by flow cytometry (Figure 2B). Next, DbTE triggered T-cell mediated cytotoxicity was assessed by the LDH assay. Dose-dependent lysis of 293T-GPNMB mediated by DbTE 87 and 293T-VCAM-1 triggered by DbTE 1B2 was observed at the E/T ratio of 10:1 (Figure 2C). Nonspecific killing of GPNMB negative 293T cells by DbTE 87 was only observed at the highest concentration of DbTE, and no nonspecific killing was observed with the lower concentrations (Figure 2D). It should be noted that DbTE 1B2 exhibited a low level of killing effect against 293T cells, which is consistent to our Western blotting data showing that 293T cells intrinsically express a low level of VCAM-1 despite no expression detected by flow cytometry (Figures 2D and 1A). These results indicate that GPNMB or VCAM-1 targeted killing was indeed triggered by their DbTEs. Similar lysis was also found in GPNMB positive sk-mel-28 cancer cells and VCAM-1 positive HuT-78 cancer cells, respectively (Figure 2E,F). However, the lysis of the both DbTEs was lower on the cancer cell lines than on the overexpressing 293T stable cell line. This may mainly be due to the expression level of target molecules on the different cell lines.

In summary, these data support the utility of targeting GPNMB or VCAM-1 expressing tumor cells by these two variable domain antibodies.

## 4 Discussion

GPNMB and VCAM-1 are both potentially powerful targets in the treatment of several types of cancer because of their high expression and function in metastasis. High expression of both GPNMB and VCAM-1 has been associated with poor prognosis in patients, indicating a need for immunotherapies targeting these proteins on cancer cells. Currently, only one ADC targeting GPNMB named CDX-011 has been conducted in phase II or phase 2b clinical trial^7,28−30^ and shown to be well-tolerated in melanoma, osteosarcoma, or triple-negative breast cancer patients, but antitumor activity is still limited. In solid cancer therapeutic development, the full length antibody still facing the limitations for tumor penetration. The antibody fragments especially variable domain antibodies showed advances since the two fragment antibody, blinatumomab^31^ and caplacizumab,^32^ were approved.

In this study, we selected and characterized two fully human V_H_ domain antibodies that target human GPNMB and VCAM-1 separately. Both antibodies showed high *K*_D_ affinities in the nanomolar range. The avidities of these two antibodies were further enhanced after converting to V_H_-Fc (Table 1). Detection of the killing effects of the two antibody-based DbTEs showed specific cell killing effects on their respective target-expressing cancer cells, demonstrating their potential for use as immunotherapies. DbTE 87 showed a specific killing against GPNMB-positive cells, and no off-target killing effects were observed (Figure 2). The moderate killing of DbTE 1B2 against 293T cells is probably due to the low intrinsic expression of VCAM-1 on 293T cells. One may note that the killing effects of DbTEs on cancer cell lines were not a standard dose-dependent curve. Further characterizations for optimize the concentration of DbTE are needed. These findings warrant further characterizations of their in vivo toxicity, specificity, and efficacy on cancer inhibition for more accurately investigate the curative effects of DbTE.

Moreover, recent studies have shown that GPNMB and VCAM-1 are associated with age-related diseases such as Alzheimer’s disease, Parkinson’s disease, and age-related neurodegeneration.^33,34^ With further development, these antibodies may be promising therapy candidates for such aging-related diseases. Taken together, the described anti-GPNMB antibody and anti-VCAM-1 antibody show significant potential as variable domain antibody-based immunotherapy candidates for diseases associated with the elevated expression of GPNMB and VCAM-1.

## Data Availability

The original contributions presented in the study are included in the article. Further inquiries can be directed to the corresponding authors.

## References

[ref1] Tse K. F.; Jeffers M.; Pollack V. A.; McCabe D. A.; Shadish M. L.; Khramtsov N. V.; Hackett C. S.; Shenoy S. G.; Kuang B.; Boldog F. L.; MacDougall J. R.; Rastelli L.; Herrmann J.; Gallo M.; Gazit-Bornstein G.; Senter P. D.; Meyer D. L.; Lichenstein H. S.; LaRochelle W. J. CR011, a fully human monoclonal antibody-auristatin E conjugate, for the treatment of melanoma. Clin. Cancer Res. 2006, 12, 1373–1382. 10.1158/1078-0432.CCR-05-2018. 16489096

[ref2] Fiorentini C.; Bodei S.; Bedussi F.; Fragni M.; Bonini S. A.; Simeone C.; Zani D.; Berruti A.; Missale C.; Memo M.; Spano P.; Sigala S. GPNMB/OA protein increases the invasiveness of human metastatic prostate cancer cell lines DU145 and PC3 through MMP-2 and MMP-9 activity. Exp. Cell Res. 2014, 323, 100–111. 10.1016/j.yexcr.2014.02.025. 24589892

[ref3] Oyewumi M. O.; Manickavasagam D.; Novak K.; Wehrung D.; Paulic N.; Moussa F. M.; Sondag G. R.; Safadi F. F. Osteoactivin (GPNMB) ectodomain protein promotes growth and invasive behavior of human lung cancer cells. Oncotarget. 2016, 7, 13932–13944. 10.18632/oncotarget.7323. 26883195 PMC4924689

[ref4] Li Y. N.; Zhang L.; Li X. L.; Cui D. J.; Zheng H. D.; Yang S. Y.; Yang W. L. Glycoprotein nonmetastatic B as a prognostic indicator in small cell lung cancer. APMIS. 2014, 122, 140–146. 10.1111/apm.12107. 23656629

[ref5] Zhang Y. X.; Qin C. P.; Zhang X. Q.; Wang Q. R.; Zhao C. B.; Yuan Y. Q.; Yang J. G. Knocking down glycoprotein nonmetastatic melanoma protein B suppresses the proliferation, migration, and invasion in bladder cancer cells. Tumour Biol. 2017, 39, 1010428317699119 10.1177/1010428317699119. 28443476

[ref6] Rose A. A.; Grosset A. A.; Dong Z.; Russo C.; Macdonald P. A.; Bertos N. R.; St-Pierre Y.; Simantov R.; Hallett M.; Park M.; Gaboury L.; Siegel P. M. Glycoprotein nonmetastatic B is an independent prognostic indicator of recurrence and a novel therapeutic target in breast cancer. Clin. Cancer Res. 2010, 16, 2147–2156. 10.1158/1078-0432.CCR-09-1611. 20215530

[ref7] Yardley D. A.; Weaver R.; Melisko M. E.; Saleh M. N.; Arena F. P.; Forero A.; Cigler T.; Stopeck A.; Citrin D.; Oliff I.; Bechhold R.; Loutfi R.; Garcia A. A.; Cruickshank S.; Crowley E.; Green J.; Hawthorne T.; Yellin M. J.; Davis T. A.; Vahdat L. T. EMERGE: A Randomized Phase II Study of the Antibody-Drug Conjugate Glembatumumab Vedotin in Advanced Glycoprotein NMB-Expressing Breast Cancer. J. Clin Oncol. 2015, 33, 1609–1619. 10.1200/JCO.2014.56.2959. 25847941

[ref8] Kuan C. T.; Wakiya K.; Dowell J. M.; Herndon J. E. 2nd; Reardon D. A.; Graner M. W.; Riggins G. J.; Wikstrand C. J.; Bigner D. D. Glycoprotein nonmetastatic melanoma protein B, a potential molecular therapeutic target in patients with glioblastoma multiforme. Clin. Cancer Res. 2006, 12, 1970–1982. 10.1158/1078-0432.CCR-05-2797. 16609006

[ref9] Maric G.; Annis M. G.; Dong Z.; Rose A. A.; Ng S.; Perkins D.; MacDonald P. A.; Ouellet V.; Russo C.; Siegel P. M. GPNMB cooperates with neuropilin-1 to promote mammary tumor growth and engages integrin alpha5beta1 for efficient breast cancer metastasis. Oncogene. 2015, 34, 5494–5504. 10.1038/onc.2015.8. 25772243

[ref10] Chung J. S.; Tamura K.; Cruz P. D. Jr; Ariizumi K. DC-HIL-expressing myelomonocytic cells are critical promoters of melanoma growth. J. Invest Dermatol. 2014, 134, 2784–2794. 10.1038/jid.2014.254. 24936834 PMC4199867

[ref11] Rose A. A.; Pepin F.; Russo C.; Abou Khalil J. E.; Hallett M.; Siegel P. M. Osteoactivin promotes breast cancer metastasis to bone. Mol. Cancer Res. 2007, 5, 1001–1014. 10.1158/1541-7786.MCR-07-0119. 17951401

[ref12] Han C. L.; Chen X. R.; Lan A.; Hsu Y. L.; Wu P. S.; Hung P. F.; Hung C. L.; Pan S. H. N-glycosylated GPNMB ligand independently activates mutated EGFR signaling and promotes metastasis in NSCLC. Cancer Sci. 2021, 112, 1911–1923. 10.1111/cas.14872. 33706413 PMC8088973

[ref13] Son S.; Kim H.; Lim H.; Lee J. H.; Lee K. M.; Shin I. CCN3/NOV promotes metastasis and tumor progression via GPNMB-induced EGFR activation in triple-negative breast cancer. Cell Death Dis. 2023, 14, 81 10.1038/s41419-023-05608-3. 36737605 PMC9898537

[ref14] Saade M.; Araujo de Souza G.; Scavone C.; Kinoshita P. F. The Role of GPNMB in Inflammation. Front Immunol. 2021, 12, 674739 10.3389/fimmu.2021.674739. 34054862 PMC8149902

[ref15] Agassandian M.; Tedrow J. R.; Sembrat J.; Kass D. J.; Zhang Y.; Goncharova E. A.; Kaminski N.; Mallampalli R. K.; Vuga L. J. VCAM-1 is a TGF-beta1 inducible gene upregulated in idiopathic pulmonary fibrosis. Cell Signal. 2015, 27, 2467–2473. 10.1016/j.cellsig.2015.09.003. 26386411 PMC4684430

[ref16] Tichet M.; Prod’Homme V.; Fenouille N.; Ambrosetti D.; Mallavialle A.; Cerezo M.; Ohanna M.; Audebert S.; Rocchi S.; Giacchero D.; Boukari F.; Allegra M.; Chambard J. C.; Lacour J. P.; Michiels J. F.; Borg J. P.; Deckert M.; Tartare-Deckert S. Tumour-derived SPARC drives vascular permeability and extravasation through endothelial VCAM1 signalling to promote metastasis. Nat. Commun. 2015, 6, 6993 10.1038/ncomms7993. 25925867

[ref17] Chen C.; Zhang Q.; Liu S.; Parajuli K. R.; Qu Y.; Mei J.; Chen Z.; Zhang H.; Khismatullin D. B.; You Z. IL-17 and insulin/IGF1 enhance adhesion of prostate cancer cells to vascular endothelial cells through CD44-VCAM-1 interaction. Prostate. 2015, 75, 883–895. 10.1002/pros.22971. 25683512 PMC4405436

[ref18] Chen Q.; Zhang X. H.; Massague J. Macrophage binding to receptor VCAM-1 transmits survival signals in breast cancer cells that invade the lungs. Cancer Cell. 2011, 20, 538–549. 10.1016/j.ccr.2011.08.025. 22014578 PMC3293160

[ref19] Wu X.; Giobbie-Hurder A.; Liao X.; Lawrence D.; McDermott D.; Zhou J.; Rodig S.; Hodi F. S. VEGF Neutralization Plus CTLA-4 Blockade Alters Soluble and Cellular Factors Associated with Enhancing Lymphocyte Infiltration and Humoral Recognition in Melanoma. Cancer Immunol Res. 2016, 4, 858–868. 10.1158/2326-6066.CIR-16-0084. 27549123 PMC5050160

[ref20] Zhang D.; Bi J.; Liang Q.; Wang S.; Zhang L.; Han F.; Li S.; Qiu B.; Fan X.; Chen W.; Jiao H.; Ye Y.; Ding Y. VCAM1 Promotes Tumor Cell Invasion and Metastasis by Inducing EMT and Transendothelial Migration in Colorectal Cancer. Front Oncol. 2020, 10, 1066 10.3389/fonc.2020.01066. 32793471 PMC7390920

[ref21] Huang J.; Zhang J.; Li H.; Lu Z.; Shan W.; Mercado-Uribe I.; Liu J. VCAM1 expression correlated with tumorigenesis and poor prognosis in high grade serous ovarian cancer. Am. J. Transl Res. 2013, 5, 336–346. 23634244 PMC3633976

[ref22] Duzagac F.; Inan S.; Ela Simsek F.; Acikgoz E.; Guven U.; Khan S. A.; Rouhrazi H.; Oltulu F.; Aktug H.; Erol A.; Oktem G. JAK/STAT pathway interacts with intercellular cell adhesion molecule (ICAM) and vascular cell adhesion molecule (VCAM) while prostate cancer stem cells form tumor spheroids. J. BUON. 2015, 20, 1250–1257. 26537072

[ref23] Wang S. S.; Yan Y. S.; Ho K. US FDA-approved therapeutic antibodies with high-concentration formulation: summaries and perspectives. Antib Ther. 2021, 4, 262–272. 10.1093/abt/tbab027. 34909579 PMC8664682

[ref24] Ruiz-Lopez E.; Schuhmacher A. J. Transportation of Single-Domain Antibodies through the Blood-Brain Barrier. Biomolecules. 2021, 11, 11 10.3390/biom11081131. PMC8394617 34439797

[ref25] Chu X.; Sun Z.; Baek D. S.; Li W.; Mellors J. W.; Shapiro S. D.; Dimitrov D. S. Human Antibody Domains and Fragments Targeting Neutrophil Elastase as Candidate Therapeutics for Cancer and Inflammation-Related Diseases. Int. J. Mol. Sci. 2021, 22, 22 10.3390/ijms222011136. PMC8539514 34681796

[ref26] Santich B. H.; Park J. A.; Tran H.; Guo H. F.; Huse M.; Cheung N. V. Interdomain spacing and spatial configuration drive the potency of IgG-[L]-scFv T cell bispecific antibodies. Sci. Transl Med. 2020, 12, 12 10.1126/scitranslmed.aax1315. PMC7437947 32161106

[ref27] Chu X.; Baek D.-S.; Li W.; Shyp T.; Mooney B.; Hines M. G.; Morin G. B.; Sorensen P. H.; Dimitrov D. S. Human antibodies targeting ENPP1 as candidate therapeutics for cancers. Frontiers in Immunology. 2023, 14, 14 10.3389/fimmu.2023.1070492. PMC9905232 36761762

[ref28] Kopp L. M.; Malempati S.; Krailo M.; Gao Y.; Buxton A.; Weigel B. J.; Hawthorne T.; Crowley E.; Moscow J. A.; Reid J. M.; Villalobos V.; Randall R. L.; Gorlick R.; Janeway K. A. Phase II trial of the glycoprotein non-metastatic B-targeted antibody-drug conjugate, glembatumumab vedotin (CDX-011), in recurrent osteosarcoma AOST1521: A report from the Children’s Oncology Group. Eur. J. Cancer. 2019, 121, 177–183. 10.1016/j.ejca.2019.08.015. 31586757 PMC6952063

[ref29] Hasanov M.; Rioth M. J.; Kendra K.; Hernandez-Aya L.; Joseph R. W.; Williamson S.; Chandra S.; Shirai K.; Turner C. D.; Lewis K.; Crowley E.; Moscow J.; Carter B.; Patel S. A Phase II Study of Glembatumumab Vedotin for Metastatic Uveal Melanoma. Cancers (Basel). 2020, 12, 12 10.3390/cancers12082270. PMC7465139 32823698

[ref30] Vahdat L. T.; Schmid P.; Forero-Torres A.; Blackwell K.; Telli M. L.; Melisko M.; Mobus V.; Cortes J.; Montero A. J.; Ma C.; Nanda R.; Wright G. S.; He Y.; Hawthorne T.; Bagley R. G.; Halim A. B.; Turner C. D.; Yardley D. A. Glembatumumab vedotin for patients with metastatic, gpNMB overexpressing, triple-negative breast cancer (″METRIC″): a randomized multicenter study. NPJ. Breast Cancer. 2021, 7, 57 10.1038/s41523-021-00244-6. 34016993 PMC8137923

[ref31] Jen E. Y.; Xu Q.; Schetter A.; Przepiorka D.; Shen Y. L.; Roscoe D.; Sridhara R.; Deisseroth A.; Philip R.; Farrell A. T.; Pazdur R. FDA Approval: Blinatumomab for Patients with B-cell Precursor Acute Lymphoblastic Leukemia in Morphologic Remission with Minimal Residual Disease. Clin. Cancer Res. 2019, 25, 473–477. 10.1158/1078-0432.CCR-18-2337. 30254079

[ref32] Duggan S. Caplacizumab: First Global Approval. Drugs. 2018, 78, 1639–1642. 10.1007/s40265-018-0989-0. 30298461 PMC6280848

[ref33] Suda M.; Shimizu I.; Katsuumi G.; Yoshida Y.; Hayashi Y.; Ikegami R.; Matsumoto N.; Yoshida Y.; Mikawa R.; Katayama A.; Wada J.; Seki M.; Suzuki Y.; Iwama A.; Nakagami H.; Nagasawa A.; Morishita R.; Sugimoto M.; Okuda S.; Tsuchida M.; Ozaki K.; Nakanishi-Matsui M.; Minamino T. Senolytic vaccination improves normal and pathological age-related phenotypes and increases lifespan in progeroid mice. Nature Aging. 2021, 1, 1117–1126. 10.1038/s43587-021-00151-2. 37117524

[ref34] Yousef H.; Czupalla C. J.; Lee D.; Chen M. B.; Burke A. N.; Zera K. A.; Zandstra J.; Berber E.; Lehallier B.; Mathur V.; Nair R. V.; Bonanno L. N.; Yang A. C.; Peterson T.; Hadeiba H.; Merkel T.; Korbelin J.; Schwaninger M.; Buckwalter M. S.; Quake S. R.; Butcher E. C.; Wyss-Coray T. Aged blood impairs hippocampal neural precursor activity and activates microglia via brain endothelial cell VCAM1. Nat. Med. 2019, 25, 988–1000. 10.1038/s41591-019-0440-4. 31086348 PMC6642642

